# A pitfall in the interpretation of plain abdnominal radiographs in neonatal intestinal perforation: a case report

**DOI:** 10.1186/1752-1947-2-335

**Published:** 2008-10-28

**Authors:** Martin J Gillies, Moti M Chowdhury, Kokila Lakhoo

**Affiliations:** 1 Children's Hospital Oxford, John Radcliffe Hospital, Headley Way, Oxford, OX3 9DZ, UK

## Abstract

**Introduction:**

The recognition of neonatal intestinal perforation relies on identification of free gas in the peritoneum on plain abdominal radiographs and the associated clinical signs. The neonatal bowel takes several hours to fill with gas, potentially obscuring one of the radiological signs of bowel perforation in the neonate.

**Case presentation:**

We describe the case of a male, Caucasian neonate, born prematurely at 35^+2 ^weeks of gestation, who was suspected before birth to be at risk of intestinal perforation, based on antenatal ultrasound signs of bowel obstruction. However, the diagnosis of intestinal perforation after birth was initially delayed because the first abdominal radiograph, requested by the neonatal team, was taken too early in the clinical progression of the neonate's condition. As a consequence, this delayed referral to the paediatric surgical team and definitive management.

**Conclusion:**

This case illustrates how consideration of the timing of abdominal radiographs in suspected intestinal perforation in the neonate may avoid misinterpretation of radiographic signs, thereby avoiding delays in referral and treatment in the crucial first few hours of life.

## Introduction

Abdominal distension with bile-stained vomiting in a neonate can potentially represent bowel obstruction. Neonatal bowel obstruction can be complicated by intestinal perforation, hence the necessity for early surgical referral in cases of neonatal bowel obstruction. Intestinal perforation may occur before birth. The preliminary imaging modality of choice to diagnose suspected bowel obstruction and intestinal perforation in a neonate is a plain supine abdominal radiograph. Accurate diagnosis often relies on recognition of abnormal gas patterns within the abdomen. However, the passage of gas through the neonatal intestine takes a number of hours after birth.

Peritoneal calcification is indicative of *in utero *bowel perforation, which can be associated with postnatal meconium peritonitis. Meconium peritonitis can be treated operatively or non-operatively, since *in utero *bowel perforations may heal spontaneously. A recent meta-analysis of the literature has helped to clarify the features of meconium peritonitis that are associated with a need for operative management [1]. Cases of meconium peritonitis were divided into four prognostic groups based on ultrasonographic criteria. Children with isolated intra-abdominal calcification had an excellent prognosis, normally avoiding surgery. The presence of one of ascites, pseudocyst or bowel dilatation had a good prognosis but an increased risk of surgery (52% required laparotomy). Having two or more of these features placed the neonate at higher risk of surgery (80% required laparotomy), whilst having all of these features was associated with the worst prognosis, this group being the only one associated with a risk of death in this study (6%). Intestinal perforation, in contrast, is associated with a higher risk of death, usually requiring early surgical intervention [2].

## Case presentation

A premature (35^+2 ^week) Caucasian neonate with suspected antenatal bowel obstruction presented to the neonatal unit with mild abdominal distension and bile stained aspirates. The infant was otherwise well at birth. No other abnormal features were detected on examination. Prenatal test for cystic fibrosis was negative.

**Figure 1:**
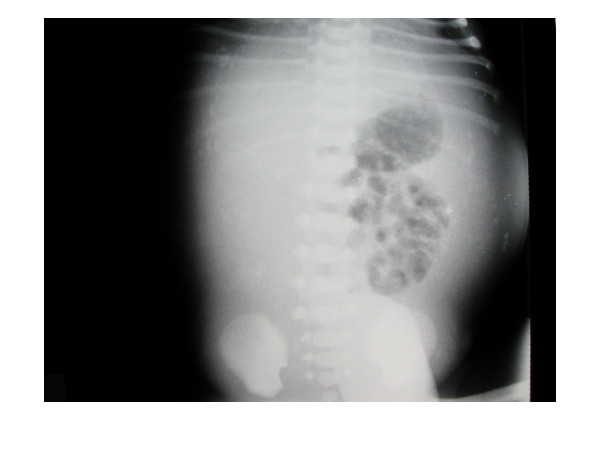
Abdominal radiograph taken 1 hour after birth, shows mottled calcification in the right upper quadrant, curvilinear calcification in the left flank and several loops of non-dilated bowel, but no free gas.

An abdominal radiograph was taken by the neonatal team within 1 hour of birth. No dilated loops were demonstrated. A calcified rim was present in the centre of the abdomen, with some irregular calcification in the right upper quadrant. There was no evidence of free gas within the peritoneal cavity (Figure 1). A diagnosis of meconium peritonitis was made on the basis of the radiographic appearance and clinical condition of the neonate. Ultrasound examination of the abdomen was not performed.

**Figure 2:**
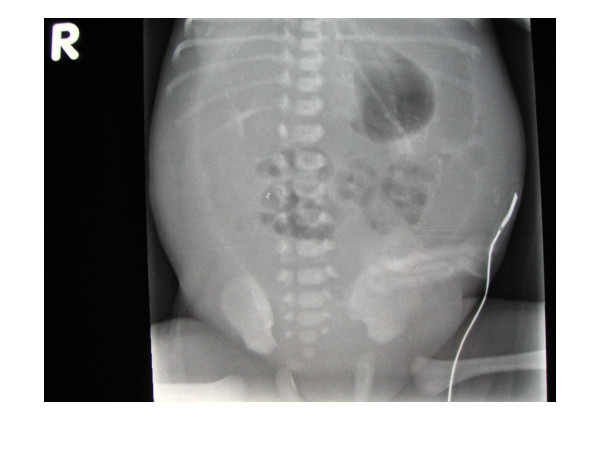
**Abdominal radiograph taken 4 hours after birth demonstrating free gas in the abdomen.** The infant had not been artificially ventilated at any point pre-operatively.

A second radiograph was performed approximately 4 hours after birth because of increasing abdominal distension in the neonate. On this second radiograph, free gas was visible in the peritoneal cavity (Figure 2). The surgical team was subsequently contacted.

At laparotomy, a large perforation was found in the ascending colon. Meconium and calcified material were drained from the peritoneal cavity. A drain was inserted and a stoma formed from the proximal ascending colon. The neonate made a good recovery postoperatively. Hirschprung's disease and cystic fibrosis were excluded as causes of the perforation.

## Discussion

The relevance of this case study to the management of neonates with suspected antenatal bowel obstruction is that the first abdominal radiograph placed our neonate in the lowest risk category, suggesting the neonate could be safely managed non-operatively. Conversely, the second abdominal radiograph, demonstrating perforation, placed our neonate in a category that generally requires surgical management [1,2].

A second issue highlighted in this patient is that this neonate was exposed to an extra dose of radiation that ultimately proved to obscure the diagnosis, therefore it was of no benefit to the patient. A recent article in the *British Medical Journal *highlighted the potential dangers of iatrogenic radiation, stressing the imperative for doctors to protect patients from unnecessary exposure [3].

## Conclusion

This study illustrates a potential pitfall in the use and interpretation of plain abdominal radiographs in the first few hours of life that may negatively impact on management of neonatal patients.

## Competing interests

The authors declare that they have no competing interests.

## Authors' contributions

MJG wrote the case study, researched and wrote the literature review. MMC critically revised the literature review and advised on radiological aspects of the case. KL was lead consultant who managed the case, instigated the conception of the case study, gave advice on the intellectual content of the case study in relation to clinical practice and obtained consent from the parents. All authors read and approved the final manuscript.

## Consent

Written informed consent was obtained from the parents of the patient for publication of this case report and accompanying images. A copy of the written consent is available for review by the Editor-in-Chief of this journal.
